# Effects of solvents and penetration enhancers on transdermal delivery of thymoquinone: permeability and skin deposition study

**DOI:** 10.1080/10717544.2018.1523256

**Published:** 2018-11-21

**Authors:** Anika Haq, Bozena Michniak-Kohn

**Affiliations:** Department of Pharmaceutics, Ernest Mario School of Pharmacy, Rutgers, The State University of New Jersey, Piscataway, NJ, USA;; Center for Dermal Research, Life Sciences Building, Rutgers, The State University of New Jersey, Piscataway, NJ, USA

**Keywords:** Thymoquinone, transdermal drug delivery, *in vitro* skin permeation, skin deposition, penetration enhancer

## Abstract

Thymoquinone (TQ) is a quinone-based phytochemical that was first identified in 1963 in *Nigella sativa* (black cumin seed) by El-Dakhakhany. Based on the ideal characteristics of transdermal delivery, TQ is potentially an attractive candidate for transdermal drug delivery. The aim of this study was to investigate the feasibility of transdermal delivery of TQ and to assess the effect of an ethanol and propylene glycol donor solvent system along with various compositions of receptor solvents. The effects of penetration enhancers on the *in vitro* skin permeation and TQ skin absorption were studied using human cadaver skin in Franz diffusion cells. The permeation of saturated solutions of TQ was investigated with 5% *v*/*v* of each of the following enhancers: Azone (laurocapram), Transcutol® P (Tc), oleic acid, ethanol, Polysorbate 80 (Tween 80), and *N*-methyl-pyrrolidone (NMP). The results indicated that Azone, oleic acid, and Tc were able to provide adequate TQ flux and may be the agents of choice for use in a novel transdermal formulation of TQ. These penetration enhancers were also able to generate TQ reservoirs in the skin that may be useful to provide sustained release of TQ from the stratum corneum over longer periods of time.

## Introduction

Alternative routes to oral drug administration such as the transdermal route are becoming even more popular in recent years. It has been predicted that the transdermal and intradermal drug delivery system market will exceed $25 billion by 2018. It was found that approximately 74% of orally administered drugs failed to exert desired pharmacological effectiveness (Marwah et al., 2016). In such cases, transdermal delivery should be considered as an alternative route to oral delivery. There are several advantages associated with transdermal drug delivery systems (TDDS). This delivery system can be very beneficial in avoiding hepatic first-pass effect (Jung et al., 2013) and gastrointestinal environment with its varying pH and a potential site for drug degradation (Singh et al., 2012b). It can also provide steady-state plasma levels, improved bioavailability, decreased side effects, and ultimately can improve patient compliance (Samad et al., 2009; Marwah et al., 2016). In 1979, FDA first approved scopolamine for the transdermal delivery system (Samad et al., 2009). Currently, only nicotine, lidocaine, estradiol, testosterone, fentanyl, nitroglycerin, etc. represent this group of TDDS (Vavrova et al., 2005). There is only a small group of drugs that are able to passively cross the skin barrier in amounts that are sufficient to give desired therapeutic concentrations and effectiveness. One limitation of success is the physicochemical properties of active molecules that are being considered for transdermal delivery. An ideal candidate for transdermal delivery should (a) have relatively low molecular weight (<500 gmol^−1^) (b) optimum lipophilicity (log *P* = 1–3), and (c) a low melting point (<200 °F).

There is also an increasing interest in the development of bioactive compounds isolated from natural products as potential drugs (Nair et al., 2010). Poor oral bioavailability is often a problem and this has diminished the potential of using drugs of natural origin (Akhondzadeh & Abbasi, 2006), thus there is a scope for alternative routes of drug administration such as transdermal for natural drugs. Thymoquinone (TQ) is a quinone-based phytochemical that was first identified in 1963 in *Nigella sativa* (black cumin seed) by El-Dakhakhany (Schneider-Stock et al., 2014). Black seed has been listed by US-FDA on its GRAS (Generally Recognized As Safe) list (Singh et al., 2012a). This is one of the most widely studied plants and for more than 2000 years the seed has been used to treat various maladies (Gali-Muhtasib et al., 2006). The seeds contain volatile oils, proteins, alkaloids, saponins, crude fiber, minerals, and vitamins (calcium, potassium, iron, sodium, zinc, vitamin A, vitamin B, vitamin B2, vitamin C, etc.), linoleic acid, oleic acid, etc. (Gali-Muhtasib et al., 2006; Deepak et al., 2011). TQ is a yellow crystalline molecule and is the main constituent of (30–48%) *Nigella sativa* extract (Singh et al., 2012a). It exhibits antioxidant, anti-inflammatory, and anti-neoplastic properties and has been studied for the treatment of cancer and various neurodegenerative diseases such as Alzheimer’s and Parkinson’s disease (Alhebshi et al., 2013). Based on the ideal characteristics required of a compound for transdermal delivery, TQ can be an attractive candidate for TDDS. It is lipophilic (log *P* = 2.54), has a low molecular weight (164.2 gmol^−1^) and a low melting point (44–45 °C). In contrast, due to its lipophilic character, TQ is not an ideal candidate for tablet or capsule formulations. Hence, one approach that has the potential of being successful is a transdermal formulation of TQ.

Skin barrier properties are mainly regulated by the stratum corneum that is primarily composed of multiple layers of keratin-rich corneocytes (the ‘bricks’) surrounded by lipid lamellae in a bilayer form (the ‘mortar’). This form is the major barrier to the delivery of drugs through the skin (Mitragotri et al., 2011; Elias, 2012). There are several strategies to overcome this skin barrier to successfully deliver active molecules. They include the use of chemical permeation enhancers, iontophoresis, electroporation, microneedles, ultrasound, magnetophoresis, photomechanical waves, etc. (Barry, 2001). The use of chemical enhancers reversibly decreases skin barrier function by disrupting intercellular stratum corneum lipids (Tiwary et al., 2007; Shah et al., 2012; Atef & Altuwaijri, 2018). Many chemicals have been investigated as penetration enhancers, such as surfactants, *N*-methyl-2-pyrrolidone, terpenes, Transcutol^®^ P, azone, and oleic acid (Williams & Barry, 2012).

The objective of this study was to investigate the feasibility of transdermal delivery of TQ. To the best of our knowledge, this is the first study to investigate both transdermal flux and skin deposition of TQ and the various conditions that may influence these parameters including vehicle/solvents, receiver composition, and permeation enhancers. This study will provide useful data for the development of novel TQ topical and/or transdermal formulations.

## Materials and methods

### Materials

Polysorbate 80 (Tween 80), *N*-methyl-Pyrrolidone (NMP), propylene glycol (PG), TQ, high-performance liquid chromatography (HPLC) grade water, and acetonitrile were purchased from Sigma–Aldrich Co. (St. Louis, MO). Ethanol was purchased from Decon Labs, Inc. (King of Prussia, PA) and Azone (laurocapram) was purchased from BOC Sciences (Shirley, NY). Phosphate-buffered saline tablets (PBS, pH 7.4) were purchased from MP Biomedicals, LLC (Solon, OH). Dermatomed human cadaver skin from the posterior torso (Female, aged 69) was obtained from New York Firefighter Skin Bank (New York, NY). Oleic Acid and Transcutol^®^ P was a gift from Croda (Edison NJ) and Gattefosse (Paramus NJ), respectively.

### Solubility determination

Saturated solutions of TQ were prepared by adding an excess amount of TQ in 5 mL of different solvents with or without 5% of penetration enhancers in well-closed amber containers. Using a Julabo SW22 shaker (Julabo USA Inc., Allentown, PA) all the amber containers with TQ in their respective solvents were agitated at 37 °C for 48 h. After 48 h all the samples were filtered through a 0.2 µm PES syringe filter media with polypropylene housing. All experiments were performed in triplicate and the drug content was measured using HPLC after appropriate dilution.

### *In vitro* skin permeation test (IVPT) studies

The skin permeability of TQ was studied *in vitro* by using Franz diffusion cells (Permegear Inc., Hellertown, PA). Dermatomed (∼500 µm) human cadaver skin with the dermal side in contact with receptor compartment was mounted on Franz diffusion cells. The donor/diffusion area was 0.64 cm^2^ and the receptor compartment was filled with PBS (pH 7.4). The precise volume of PBS that was needed to fill the receptor compartment was measured for each cell and was included into the calculations. Before using the skin, the samples were slowly thawed, cut into appropriate pieces and then soaked in filtered PBS (pH 7.4) for 15 minutes. The diffusion cells were allowed to equilibrate at 37 °C for 15 minutes. Once reached equilibrium, at time zero the donor compartment was filled with 0.5 mL of saturated solution of TQ in PG/ethanol vehicle with or without 5% of each enhancer used in this study. The receptor compartment of each cell was maintained at 37 °C under synchronous continuous stirring using a magnetic stirrer at 600 rpm. At each time point (3, 4, 6, 8, 10, 12, and 24 h) 300 µL of receptor samples were withdrawn from the sampling port and were immediately replaced with an equal volume of PBS (pH 7.4). At the end of 24 h, receptor aliquots of 300 µL were then analyzed using a validated HPLC method described below.

### High-performance liquid chromatography (HPLC)

TQ was quantified using a validated HPLC method by Agilent 1100 series HPLC instrumentation (Agilent Technologies, Santa Clara, CA) coupled with UV detection (Diode array detector – DAD). A mobile phase of 80% Acetonitrile and 20% water was pumped through an Agilent Eclipse XDB-C18 5 µm, 250 × 4.6 mm column at a flow rate of 1.0 mL/minute, and an injection volume of 20 μL was applied to analyze each sample. The column temperature was set to 23 °C with UV detection of 250 nm. The retention time for TQ was 4.2 minutes. The method was linear at a concentration of 0.39–100 µg/mL with *R* ^2^ value of 0.99. Intra- and inter-day precision and accuracy of the method showed a %CV of 0.01 and 0.1, respectively, which is lower than the requirements of 2%.

### Determination of TQ concentration in the skin

At the end of the 24 h of permeation study, the left-over donor solutions and donor compartments were carefully transferred into 50 mL centrifuge tubes. The skin surface was then patted dry with a cotton swab. Each Franz diffusion cell with the skin still mounted on it was held in slightly tilted position over the 50 mL centrifuge tube and 1 mL of ethanol was placed dropwise to wash the skin surface and again a few cotton swabs were used to pat the skin dry. All the cotton swabs with an additional 4 mL of ethanol were placed in 50 mL centrifuge tubes and prepared for HPLC analysis. The skin was then removed from the diffusion cell and was cut around the diffusional area, air dried, and accurately weighed. The samples were then cut into very small pieces and then placed into bead bug tubes. The tubes were then filled with 1 mL ethanol and were homogenized for 9 minutes (3 min of 3 cycles) by using bead bug homogenizer (BeadBug^TM^ Microtube homogenizer, D1030 (Benchmark Scientific, Sayreville, NJ)). All the skin and donor samples were then placed in a Julabo SW22 shaker and were agitated at 37 °C for 24 h. After 24 h all the skin samples were centrifuged at 1200 rpm for 5 minutes and were filtered through a 0.45 µm polypropylene filter media with polypropylene housing. TQ concentrations were expressed as ng of TQ per skin weight in mg.

### Data and statistical analysis

The flux of TQ was determined from the slope of the linear portion of the cumulative amount of TQ permeated per unit skin surface area (µg/cm^2^) versus time (h) plot. Individual permeation profiles were generated to calculate average TQ flux with respective standard deviation. Permeability coefficient (*K*_p_) and the effectiveness of the penetration enhancers (ER = enhancement ratio) were determined using Equations 1 and 2, respectively,

$$K_{p}=\text{Flux}/\text{solubility}$$

(1) (Sloan et al., 1986)

(2) ER=Flux with the enhancer/flux without the enhancer

The lag time was calculated from extrapolation of the linear portion to the *x*-axis intercept of the permeation profile. Results are reported as mean ± SD (*n* = 5). The statistical analysis of the data was performed by using one-way ANOVA and Student’s *t*-test, and *p* values < .05 were considered significant.

## Results and discussion

### Thymoquinone solubility study

There is little or no literature available on TQ solubility in commonly used vehicles (Salmani et al., 2014). The saturation solubility of TQ determined experimentally in several commonly used solvents is provided in Table 1. The results show that highest TQ solubility can be achieved with ethanol (19 ± 2.6 mg/ml). Adding water to ethanol in 1:1 ratio significantly decreased (*p* < .0002) the solubility of TQ (0.42 ± 0.06 mg/ml). Although TQ has good solubility in PBS pH 7.4 (0.66 ± 0.01 mg/ml), it can be further increased to 0.79 ± 0.03 mg/ml by adding ethanol to PBS pH 7.4 in 1:1 ratio. Thus, we can conclude that ethanol synergistically increases TQ solubility when used with a co-solvent. In contrast, water reduced TQ solubility when used with another solvent such as ethanol or methanol.

**Table 1.** Summary of the solubility study results.

Solvents	Solubility (mg/mL) ± SD
Methanol	0.4 ± 0.02
Ethanol	19 ± 2.6
Ethanol:water (1:1)	0.42 ± 0.06
Methanol:water (1:1)	0.34 ± 0.03
Propylene glycol	9.7 ± 0.16
PBS pH 7.4	0.66 ± 0.01
Ethanol:PBS pH 7.4 (1:1)	0.79 ± 0.03
Polyethylene glycol	2.9 ± 0.2

The values represent the concentration of TQ ± SD (*N* = 3) in mg/mL at 48 h.

### Effect of propylene glycol and ethanol as donor solvents

**Figure 1.**
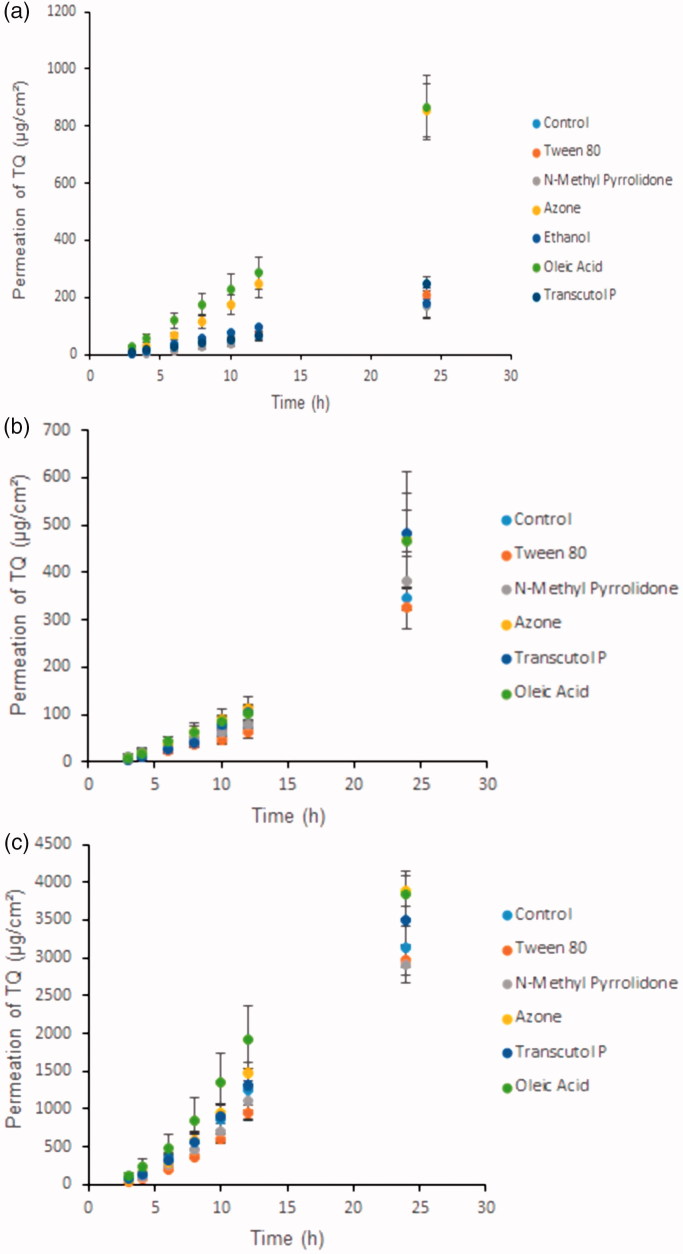
(a) Effect of different penetration enhancers in propylene glycol vehicle on the permeation of thymoquinone (µg/cm^2^) against time (h) through human cadaver skin, (b) Effect of different penetration enhancers in ethanol vehicle on the permeation of thymoquinone (µg/cm^2^) against time (h) through human cadaver skin, (c) Effect of different penetration enhancers in ethanol vehicle and ethanol: PBS (pH 7.4) receptor on the permeation of Thymoquinone (µg/cm^2^) against time (h) through human cadaver skin. Time points were measured at 3, 4, 6, 8, 10, 12, and 24 h. Each point represents the mean ± S.D. of five replicates.

Ethanol and propylene glycol are commonly used solvents for lipophilic drugs (Bendas et al., 1995). Both are important solvents and/or co-solvents for topical and transdermal formulations. Penetration parameters of TQ and the effect of 5% penetration enhancers on the solubility of TQ using PG are summarized in Table 2 and penetration parameters of TQ and the effect of 5% penetration enhancers on the solubility of TQ using ethanol are summarized in Table 3. For both solvents, the formulations were applied to the skin as saturated solutions with 5% of enhancer and the study was continued for 24 h. Figure 1(a–c) shows that 24 h was sufficient to reach steady-state concentrations in the receptor of the Franz cell. It was found that there were significant differences in the penetration parameters of TQ in the presence of various chemical enhancers and vehicles (ethanol and PG). The rank order for TQ flux with each enhancer from PG was as follows: Azone + oleic acid > Tc > control + Tween 80 > ethanol > NMP. The solubility of TQ in PG vehicle with 5% penetration enhancers was in the following rank order: ethanol > Azone > oleic acid > Tc > Tween 80 > control > NMP. In contrast, the rank order for the TQ flux with each enhancer from ethanol was as follows: Tc > oleic acid > Azone > control > Tween 80 + NMP and the solubility of TQ in ethanol vehicle with 5% penetration enhancers was in the following rank order: Tc > oleic acid > Azone > control > Tween 80 > NMP. The cumulative amount of TQ penetrated per cm^2^ from both Azone and oleic acid formulations after 24 h through human cadaver skin was 1.8-fold reduced in ethanol compared with PG. In this study, TQ solubility in PG was found to be 9.7 ± 0.16 mg/ml (Table 1), which is significantly lower (*p* < .0035) than the solubility of TQ in ethanol (19 ± 2.6 mg/ml). In addition, 5% ethanol in PG vehicle showed the highest solubility of TQ (Table 2) compared with other penetration enhancers but TQ was not able to permeate well from this formulation compared with other. The solubility and penetration parameters data showed that although ethanol was able to increase the solubility of TQ it was not able to increase the flux of permeant from the saturated solutions of TQ. This result was in agreement with studies on percutaneous absorption of aspirin conducted by Levang et al. (1999). According to this study, although aspirin showed the highest solubility in 100% ethanol vehicle the maximum flux of aspirin was obtained with 80% ethanol and 20% PG solvent. In another study obtained by Krishnaiah et al. (2002) where nicardipine hydrochloride showed higher solubility in propylene glycol compared with ethanol, the flux of the drug from propylene glycol was lower than that from ethanol (Krishnaiah et al., 2002). From the above findings, it can be postulated that the thermodynamic activity of TQ in the formulation was modified most probably by the rapid permeation and evaporation of ethanol. Moreover, different penetration enhancers and vehicles act differently due to their different mechanisms of action. It is believed that ethanol may disrupt the cutaneous barrier function by removing intercellular material (Levang et al., 1999). Ethanol is also involved in lipid fluidization and extraction (Kurihara-Bergstrom et al., 1990; Bommannan et al., 1991). In contrast, PG may increase drug permeability by solvating the α-keratin structures of the cells (Bendas et al., 1995).

**Table 2.** Penetration parameters of thymoquinone through human cadaver skin (*N* = 5) after 24 h and the effect of 5% penetration enhancers on the solubility of TQ using propylene glycol.

Formulation	TQ Flux (µg/cm²/h)	TQ *Q*_24_ (µg/cm²)	Lag time (h)	P × 10^–3^ (cm/h)±SD	Solubility (mg/mL) ± SD*
Control	11.02 ± 1.2	208 ± 23	3.17 ± 0.07	1.25 ± 0.13	8.6 ± 0.3
Tween 80	11.09 ± 1.5	208 ± 16	3.25 ± 0.4	1.03 ± 0.14	9.39 ± 1.1
NMP	9 ± 1.5^b^	167 ± 38	3.9 ± 0.3	1.07 ± 0.19	8.5 ± 0.2
Azone	49.3 ± 5.6^a^	854 ± 93	4.2 ± 0.3	3.59 ± 0.36	14.98 ± 1.4
Ethanol	10.59 ± 1	180 ± 55	2.34 ± 0.2	0.69 ± 0.06	15.72 ± 0.5
Oleic Acid	46.3 ± 4.5^a^	865 ± 113	3.2 ± 0.3	3.03 ± 0.26	13.64 ± 2.1
Transcutol^®^ P	14.23 ± 1.4^a^	247 ± 26	3.7 ± 0.2	1.37 ± 0.12	11.09 ± 0.7

a Significant increase in TQ flux (*p* < .05).

b Significant reduction in TQ flux (*p* < .05).

* The values represent the concentration of TQ ± SD (*N* = 3) in mg/mL at 48 h.

**Table 3.** Penetration parameters of thymoquinone through human cadaver skin (*N* = 5) after 24 h and the effect of 5% penetration enhancers on the solubility of TQ using ethanol.

Formulation	TQ flux (µg/cm²/h)	TQ *Q*_24_ (µg/cm²)	Lag time (h)	P × 10^–3^ (cm/h) ± SD	Solubility (mg/mL) ± SD*
Control	21.28 ± 1.8	347 ± 18	4.21 ± 0.8	0.90 ± 0.07	29.56 ± 0.1
Tween 80	20.68 ± 2.8	325 ± 43	4.8 ± 0.2	0.85 ± 0.11	23.76 ± 0.3
NMP	20.68 ± 2.8	382 ± 62	4.30 ± 0.2	0.90 ± 0.12	23.26 ± 0.7
Azone	27.97 ± 5.8^a^	468 ± 98	4.38 ± 0.3	0.97 ± 0.20	29.68 ± 1.3
Oleic Acid	28.25 ± 10^a^	466 ± 146	4.31 ± 0.6	0.97 ± 0.35	30.36 ± 1.3
Transcutol^®^ P	29.53 ± 3^a^	483 ± 48	4.93 ± 0.2	0.96 ± 0.09	31.19 ± 0.5

a Significant increase in TQ flux (*p* < .05).

* The values represent the concentration of TQ ± SD (*N* = 3) in mg/mL at 48 h.

### Effects of various penetration enhancers

Comparing the penetration parameters of the two vehicles it was found that there was a significant increase (*p* < .05) in TQ flux of control, Tween 80, and NMP formulation from ethanol. In contrast, there was a significant increase (*p* < .05) in TQ flux of Azone and oleic acid formulation from PG compared with ethanol. Such behavior of Azone and oleic acid in PG vehicle can be explained by the fact that there might be a synergistic effect between Azone and oleic acid with PG as was reported previously in the literature (Williams & Barry, 2012). A study conducted by Bennett & Barry (1985) concluded that 5% oleic acid in PG increased the penetration of both mannitol and hydrocortisone (Bennett & Barry, 1985). Our previous study (Haq et al., 2018) has demonstrated that nicotine flux was significantly greater (*p* < .05) from PG vehicle with 5% Azone compared with all other formulations containing 5% of other different penetration enhancers.

Azone is a highly lipophilic compound (log *P* = 6.2) and is the first molecule to be specifically designed as a skin penetration enhancer (Stoughton & McClure, 1983). Although the efficacy of Azone is influenced by the vehicle selection, it mostly enhances the permeability of an active molecule by interacting with the stratum corneum lipid domains (Williams & Barry, 2012). Oleic acid was also found to exert its effect by either modifying or by interacting with the stratum corneum lipid domains (Pathan & Setty, 2009).

Interestingly, 5% Tc performed better in ethanol by showing a two-fold increase in TQ flux compared with PG. It has been reported that Tc increases the solubility of drugs in the skin, but its exact mechanism of action is still unexplored (Lane, 2013). By analyzing penetration parameters of TQ in PG and ethanol it was found that 5% Azone in PG showed the highest permeation of TQ. In contrast, 5% NMP in PG showed the lowest permeation of TQ. This result is not surprising as NMP interacts with hydrophilic molecules and provides higher flux (Williams & Barry, 2012). In contrast, TQ is a highly lipophilic molecule so the interaction between NMP and TQ is not as good as with hydrophilic molecule. This finding was in consistent with that observed by Aungst et al. (1986) when they used 10% of various penetration enhancers in PG vehicle in studying the enhancement of naloxone penetration through human skin (Aungst et al., 1986). This study concluded that naloxone flux was unaffected by 10% NMP. In addition, 5% NMP in ethanol showed equal TQ permeation compared with 5% Tween-80 in ethanol. A study of the transdermal permeation of dimethyl fumarate from PG vehicle showed that different concentrations of Tween-80 either provided equal or slightly increased permeation of dimethyl fumarate compared with different concentrations of NMP used in this study (Ameen & Michniak-Kohn, 2017). Tween-80 as a nonionic surfactant has a minor penetration enhancement effect in human skin and can lower the thermodynamic activity of permeants (Lane, 2013). Although 5% ethanol in PG was not able to increase TQ flux compared with all other formulations, there was a reduction of lag time. These data support the study reported by Rao et al. (2015) and also confirmed that ethanol can reduce the time needed for a drug to reach steady state (Rao et al., 2015).

### Effect of receiver solvent composition

**Figure 2.**
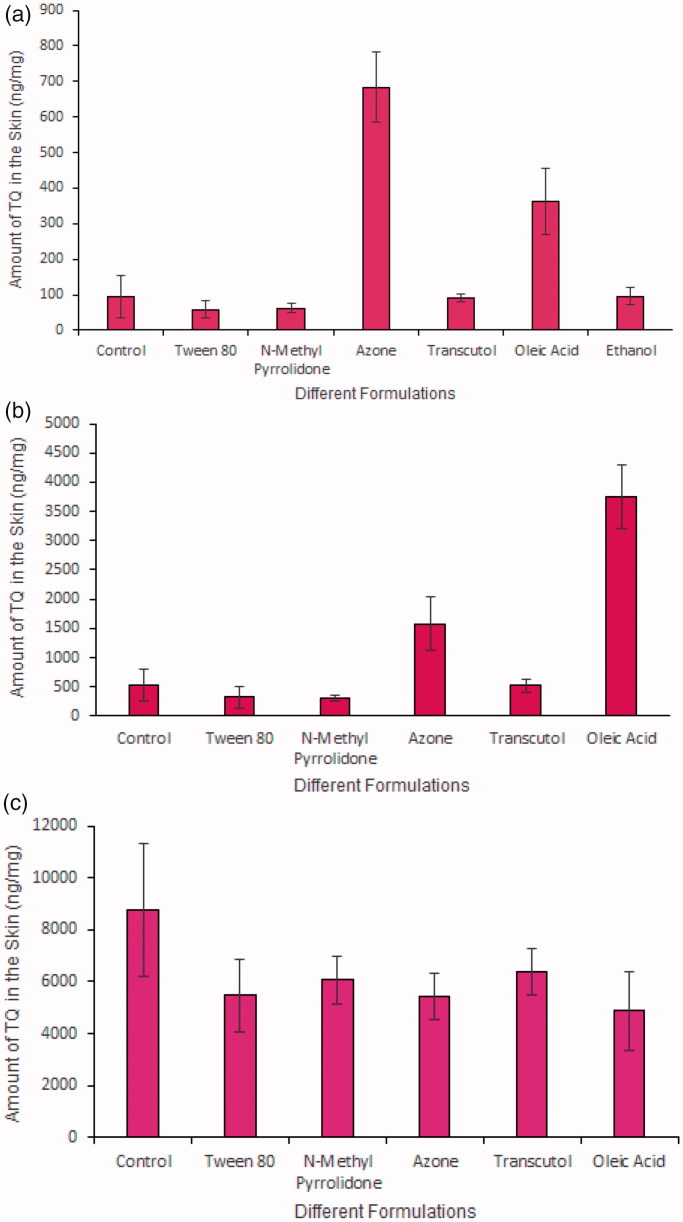
(a) Amount of TQ detected after application of different formulations containing PG vehicle and penetration enhancers for 24 h in human cadaver skin (*N* = 5, mean ± SD), (b) Amount of TQ detected after application of different formulations containing ethanol vehicle and penetration enhancers for 24 h in human cadaver skin (*N* = 5, mean ± SD), (c) Amount of TQ detected after application of different formulations containing ethanol vehicle and penetration enhancers for 24 h in human cadaver skin; receptor composition was 60:40 (Ethanol:PBS) to evaluate the pull/drag effect of ethanol vehicle in the formulation (*N* = 5, mean ± SD).

Table 4 summarizes the penetration parameters of TQ using ethanol and ethanol: PBS pH 7.4 (60:40) as receptor solvents. The rank order for the TQ flux of each enhancer from ethanol and ethanol: PBS receptor was as follows: Azone > Tc > oleic acid > Tween 80 > control > NMP. In all three permeation studies, NMP showed the lowest permeation of TQ. In contrast, Azone was a better penetration enhancer compared with all other enhancers used in this study as it provided the highest flux in PG and in ethanol: PBS receptor solvent. It was interesting to find that Tween-80 acted as a control in PG and it acted as an NMP in ethanol vehicle by providing equal TQ permeation (Tables 2 and 3). By changing the receptor composition, we were able to increase the permeability capacity of Tween-80 (Table 4) compared with control and NMP in PG and ethanol vehicle, respectively. Based on these data, this may be due to the fact that the receptor composition improved the thermodynamic activity of TQ in Tween-80 formulation as the flux is proportional to a gradient of thermodynamic activity and also due to the improvement of TQ skin reservoir (Figure 2(c)).

**Table 4.** Penetration parameters of thymoquinone through human cadaver skin (*N* = 5) after 24 h using ethanol vehicle and ethanol:PBS pH 7.4 (60:40) receptor solvents.

Formulation	TQ Flux (µg/cm²/h)	TQ *Q*_24_ (µg/cm²)	Lag time (h)	P × 10^−3^ (cm/h) ± SD	ER
Control	160 ± 5	3140 ± 270	3.6 ± 0.5	5.54 ± 0.2	
Tween 80	168 ± 11	2966 ± 188	4.3 ± 0.1	5.74 ± 0.38	1.05
NMP	155 ± 15	2918 ± 250	3.8 ± 0.5	5.30 ± 0.53	0.96
Azone	206 ± 18^a^	3885 ± 202	3.8 ± 0.4	7.26 ± 0.64	1.28
Oleic Acid	171 ± 8^a^	3839 ± 316	2.7 ± 0.8	6.04 ± 0.30	1.06
Transcutol^®^ P	177 ± 19^a^	3504 ± 353	3.4 ± 0.2	6.22 ± 0.66	1.11

ER: enhancement ratio.

a Significant increase in TQ flux (*p* < .05).

### Effect (pull or drag) of permeation enhancers and vehicle on TQ skin deposition

Both the donor and receptor solvent composition influence the capability of the permeation enhancer and vehicle to either increase or decrease the levels of TQ skin deposition. Figure 2(a) shows the amount of TQ in ng/mg (PG) detected at 24 h in human cadaver skin. The rank order for TQ skin deposition in PG using different enhancers are as follows: Azone > oleic acid > ethanol > control > Tc > NMP > Tween 80. This result shows that both Azone and oleic acid were able to provide a TQ reservoir. Moreover, with PG Azone showed almost a two-fold increase in TQ skin deposition compared with that for oleic acid. Figure 2(b) shows the amount of TQ in ng/mg (ethanol) detected after 24 h in human cadaver skin and the rank order is as follows: oleic acid > Azone > control > Tc > Tween 80 > NMP. Although oleic acid, Azone, and Tc in ethanol were not as efficient as oleic acid, Azone and Tc in PG in terms of TQ flux, but they were able to provide highest TQ skin deposition. Hence, with the ethanol, all the enhancers were able to create TQ reservoirs within the skin membrane and more TQ was deposited in the skin rather than migrating to the receptor compartment of the Franz diffusion cell. In other words, ethanol increases the capacity of the stratum corneum for drug uptake. As both vehicle and enhancer also penetrate through the skin with the active molecule there might be some pull or drag effect associated with using two different vehicles. We can further understand the pull/drag effect of ethanol vehicle on TQ skin deposition by analyzing the skin deposition study (Figure 2(c)) after changing the receptor composition to 60:40 (Ethanol:PBS). The rank order is as follows for skin deposition: control > Tc > NMP > Tween 80 > Azone > oleic acid. This skin distribution ranking profile is almost opposite comparing the skin permeation ranking profile. This time ethanol control was able to provide the highest skin reservoir compared with the other formulations with different enhancers and Azone and oleic acid showed the lowest TQ skin deposition. From the above result, it can be concluded that ethanol was able to pull more drug into the skin and as this control formulation did not contain any enhancer the ethanol was not able to further enhance the drug permeability. In contrast, all the enhancers used in this study showed low ‘pulling’ effect and they were not as efficient as the ethanol control to pull more drug into the skin membrane. Rather these enhancers (Azone, oleic acid, and Tc) showed enhanced permeation as the enhancers have permeation enhancing effect. It must be noted here, that we have analyzed the full-thickness skin for TQ skin deposition/concentration study and the skin samples were analyzed at 24 h of permeation. However, further studies should be conducted to examine TQ skin absorption and skin deposition.

## Conclusions

Skin penetration of TQ was influenced not only by the physicochemical properties of the vehicle but also by the other experimental parameters such as receiver solvent composition and permeation enhancers. This study showed donor–receiver inter-relationships governing TQ penetration and skin absorption together with effects of penetration enhancers. It can be concluded that transdermal permeation and skin deposition of TQ can be obtained by using certain penetration enhancers and vehicles. Azone, oleic acid, and Tc at a concentration of 5% *v*/*v* were able to provide measurable TQ fluxes and may be used to further develop a novel transdermal formulation of TQ. These penetration enhancers were also able to generate TQ reservoirs in the skin that might be useful in providing sustained release of TQ from the stratum corneum over longer periods of time.
